# Understanding User Requirements for a Senior-Friendly Mobile Health Application

**DOI:** 10.3390/geriatrics7050110

**Published:** 2022-10-01

**Authors:** Farzana Parveen Tajudeen, Nurhidayah Bahar, Maw Pin Tan, Mumtaz Begum Peer Mustafa, Nor Izzati Saedon, Jenifer Jesudass

**Affiliations:** 1 Department of Management, Faculty of Business and Economics, Universiti Malaya, Kuala Lumpur 50603, Malaysia; 2 Center for Software Technology and Management, Universiti Kebangsaan Malaysia, Bangi 43600, Malaysia; 3 Department of Medicine, Faculty of Medicine, Universiti Malaya, Kuala Lumpur 50603, Malaysia; 4 Department of Software Engineering, Faculty of Computer Science & Information Technology, Universiti Malaya, Kuala Lumpur 50603, Malaysia

**Keywords:** mhealth, mobile applications, older people, user requirement, usability, health management

## Abstract

The advancement of mobile technologies has motivated countries around the world to aim for smarter health management to support senior citizens. However, the use of mobile health applications (mHealth apps) among senior citizens appears to be low. Thus, drawing upon user expectations, the present study examined user requirements for a senior-friendly mHealth application. A total of 74 senior citizens were interviewed to explore the difficulties they encounter when using existing mobile apps. This study followed Nielsen’s usability model to identify user requirements from five aspects, namely learnability, efficiency, memorability, error, and satisfaction. Based on the results, a guideline was proposed pertaining to usability and health management features. This guideline offers suggestions for mHealth app issues related to phrasing, menus, simplicity, error messages, icons and buttons, navigation, and layout, among others. The study also found that speech recognition technology can help seniors access information quickly. The proposed guideline and findings offer valuable input for software and app developers in building more engaging and senior-friendly mHealth apps.

## 1. Introduction

Population ageing is occurring across many countries around the world. Notably, developing countries such as Malaysia are ageing at a far more rapid rate than developed countries, which already underwent population ageing in the previous century [1]. Indeed, senior citizens are one of the target groups in the Malaysian government’s Shared Prosperity Vision 2030, which outlines its 10-year goal to restructure the country by improving its citizens’ standard of living [2]. The report specifically mentions that senior citizens should be given high consideration, equal opportunities, and access to all resources. According to the Government of Malaysia’s official online portal [3], senior citizens are defined as those persons who are 60 years old and above. This definition aligns with the definition provided by the United Nations.

With regard to health, it is crucial for senior citizens to have access to sufficient health-related knowledge in order to lead a healthy and active lifestyle. In line with this, the World Health Organization (WHO) defines health literacy skills as the personal characteristics and social resources needed for individuals and communities to access, understand, appraise, and use information and services to make decisions about health [4,5]. About 93% of the adult population in Malaysia has limited health literacy [6]; hence, it is important to provide a platform for senior citizens to access health-related information to improve their health literacy and self-health management. In response to this, to date, the healthcare community has found the concept of mobile health to be the most ideal tool for the self-management of healthcare services, especially for.

Mobile health applications (or mHealth apps) play an important role in the health monitoring of people. However, most digital medical assistance devices tend to encounter failure due to user errors. This circumstance emerges because developers give less importance to usability attributes when creating these devices. Complicated designs and features confuse users, leading them to commit errors (e.g., pressing the wrong icon). This oversight has caused myriad difficulties for users in operating mHealth apps [7]. Senior users of mobile apps, in particular, are challenged by apps’ complicated menus [8], small text fonts [9], and other usability-related issues. Clearly, these situations occur because the apps were designed without considering their needs [10]. It is thus evident that mHealth apps need to include specific criteria that are appropriate for senior persons. Previous studies have investigated the usability attributes of mobile applications [11,12], but so far, few have focused on senior users, whose needs vary from that of the younger generation who grew up with mobile phones and touchscreen technology [13]. The objective of this study is to address this existing gap by conducting an in-depth investigation on the needs and expectations of senior citizens when they use mobile apps. In addition, it seeks to identify the type of mobile apps used by seniors, the challenges faced by them when using these apps, and their health-related information seeking and retrieval behaviour. Based on this information, the study aims to present a detailed guideline on usability attributes, features, and health management functions for mHealth apps. The proposed specifications could serve as a guidance for software and mobile app developers in building more engaging and senior-friendly mHealth apps.

### 1.1. Literature Review

#### 1.1.1. Health Literacy & Digital Literacy

Health literacy is crucial for people in all stages of life; it provides the ability to access, comprehend, evaluate, and communicate information related to one’s health, which facilitates better health management [14]. Digital technologies, including the Internet, smartphones, and mobile applications, can improve individuals’ health literacy. The availability of mobile Internet helps people access different types of health information spontaneously from several online sources. According to the American Library Association (ALA) [15], digital literacy is the ability to use technologies to find, evaluate, create and communicate information requiring not only technical skills but also cognitive skills. Digital literacy is important to survive in this digitally dominant world. Lack of digital literacy is also a barrier for adoption of many technologies. For instance, the State of Mobile Internet Connectivity Report [16] states that a lack of digital literacy or skills is the main barrier to mobile internet adoption among a third of the respondents from the participating countries. Poor digital literacy may also affect the adoption of mHealth applications among senior citizens. mHealth provides several benefits to senior citizens which are further explained in the next section.

#### 1.1.2. mHealth Applications

Hussein et al. [17] suggested that mHealth is a health practice that evolved from mobile technology to meet patients’ healthcare needs. The implementation of mHealth apps makes healthcare services more accessible, effective, and affordable. According to Statista (2022), there were about 54,603 mHealth apps available on the google play store as of Q2 2022 which is up by 4% compared to Q1 2022. Similarly, there were about 52,406 mHealth apps available on Apple app store as of Q2 2022 which is up by 2% compared to previous quarter [18,19]. Generally, available mHealth apps can be classified into several categories, such as general health and fitness, chronic illness care management, medication management, women’s health, symptom checker, healthcare professional finder, and management of clinical records, among others. Some mHealth apps were also developed to help senior people monitor and manage their health activities. Health Tracker, Health Pal, My Fitness Pal, All Well Senior Care, Senior Fitness, Senior Beginner Workout, and Care Manual are some of the general and senior-focused mHealth apps that are available globally. These apps provide tips, health information, consultations, and other related services.

#### 1.1.3. mHealth Apps in Malaysia

The Malaysian Ministry of Health (MoH), in collaboration with the Malaysian Administrative Modernisation and Management Planning Unit (MAMPU), launched an mHealth application project called myHealth KKM. This project aimed to facilitate patients in accessing health information via smartphones. There are also other mHealth apps available for Malaysians. For instance, BookDoc, Naluri, Doctor Anywhere, Doc2Us, and DoctorOnCall are some of the apps that have been developed to provide health-related services to people of all ages, not specifically to senior persons.

As mentioned earlier, the various mHealth apps that are available have the potential to provide users access to information as well as to motivate and enhance their involvement in health management. However, to deliver high-quality apps and increase their usage, the development of such apps should be based on a thorough knowledge of user needs.

#### 1.1.4. Understanding User Needs and Expectations for User-Friendly mHealth Applications

Understanding user needs is important for the success of any information system, including mobile applications. According to Cornet et al. [20], user-centred design (UCD) (also called human-centred design) is a powerful framework that can be used to develop mHealth apps that are useful, easy to use, and satisfying. UCD is a process that takes into account the end users and other stakeholders’ needs in a project life cycle, during which information is used iteratively to study, design, and review services and systems. mHealth projects benefit from UCD by utilizing feedback from patients, informal caregivers, physicians, and other stakeholders to develop better prototypes and continuously refine their strategies, thereby improving their accessibility, adoption, and future success when introduced [20,21,22,23].

Realising the importance of user-centric designs, previous researchers have focused on building mHealth apps based on user requirements. For instance, Floch et al. [24] built and evaluated an mHealth app ecosystem for the self-management of cystic fibrosis. Their paper addressed the study participants’ issues and needs in terms of the specific features in an ecosystem of self-management apps. Some of the useful features identified in the study were education, measurement of enzyme dose, diet control, treatment organisation, health diary, follow-up of treatment, realistic instructions for treatment, contact with physicians, and communication with peers.

In relation to this, Farao et al. [25] suggested an mHealth technology design paradigm that incorporates the frameworks of Information Systems Research (ISR) and design thinking. The authors illustrated the use of their proposed system in the form of a Tuberculin Skin Test (TST) app which was used to screen patients for latent tuberculosis infection. The combined framework recognises the importance of engaging users in the implementation of mHealth technologies, particularly in developing countries with insufficient resources. This notion was reinforced by Holmen et al. [26], who mentioned that to get a clearer picture of mHealth apps’ potential in the context of diabetes, it is necessary to understand user needs and expectations, as well as the factors that influence the usage of the apps. Correspondingly, Pais [27] developed a proof-of-concept prototype of a mobile application supporting women with gestational diabetes mellitus (GDM). Using the UCD approach, the developed prototype was able to meet end-user criteria and expectations, which helps retain both users and usage.

Most health and medical apps seldom retain users, thereby leaving untapped potential in terms of their ability to facilitate disease management, data sharing, and patient-provider communication. In light of this, Pan and Zhao [28] explored the feasibility of mHealth use and the factors that inhibit the adoption and continual use of mHealth technologies among 20 patients. Among the respondents, only five reported prior experience with mHealth-related applications. Of these five, only two had continued using mHealth applications on a regular basis. The reasons provided for discontinuing use include difficult interface comprehension, apps’ inability to provide effective diagnosis, and time needed for data entry. Factors such as safety risks, ease of use, and accuracy of disease prediction were also identified as concerns. It was further found that features that would enhance the usage of mHealth apps comprise medical record consolidation, convenient appointment scheduling and medication refills, integration of wearable health tracking devices, and facilitation of direct patient-to-patient and doctor-to-patient interactions.

Similarly, Santini et al. [29] examined user requirements for a Virtual Coach (VC) system to motivate senior citizens to adopt a healthy lifestyle. Data was collected from older workers aged 55 years and above through two-wave cross-national focus groups in Austria, Italy, and the Netherlands. A telephone follow-up study was also carried out with end-users. The user requirement results showed that participants desire a VC system that resembles the personality of a physical coach; that is, the system should exhibit empathic behaviour, provide motivational messages, and adopt non-directive language. Based on the above literature, it is clear that previous user requirement studies have mainly looked at users from all age groups. Only a few have focused on seniors and their usage, possibly because they are not considered major users of mobile apps. In fact, smartphone usage among senior citizens has been noted to be relatively low compared to their younger counterparts [30].

#### 1.1.5. Smartphone and mHealth App Usage by Senior Citizens

Implementing mHealth through smartphones can enhance the independence of senior citizens and assist family caregivers in care provision. mHealth apps also have the capacity to educate users and encourage them to interact and share health-related information with each other via digital platforms. Despite these benefits, the use of mHealth among senior citizens remains low [31]. Quinn et al. [31] proposed that additional education and technology usage training may be provided to senior citizens and their caregivers to increase the apps’ usability and engagement. Another approach to increase the adoption of mHealth apps among senior citizens is to understand and implement their needs as features in the apps. This would support senior citizens in their digital health management [32]. Moreover, the COVID-19 outbreak has elevated the importance of mHealth for the senior citizens. Abbaspur-Behbahani et al.’s [33] systematic review investigated the application of mHealth to support the senior citizens during outbreak. Their results showed that throughout the pandemic period, mHealth was useful to senior citizens for various purposes, such as information provision, therapy, health monitoring, and mental health consultation. More significantly, country-specific mHealth apps were developed during the COVID-19 pandemic to perform contact tracing, symptom self-assessment, and infection prevention. However, senior citizens faced several issues in using these apps due to their lack of understanding about the apps’ features and functions [34].

Therefore, realising the growing of importance of mHealth apps and the difficulty faced by senior citizens in using these apps, it is important to consider the systematic integration of senior-friendly features into mHealth apps for the benefit of senior people. Chao [35] suggested that the current framework of usability engineering can ensure mHealth apps’ user-friendliness, but not their persuasiveness. A pilot design project was conducted using a dietary management mHealth app that was developed using the UCD approach (persona, usage scenario, task review, and cognitive walkthrough). The outcome of the pilot test showed good potential for the technology’s adoption among senior persons. Morey et al. [7] assessed the usability of a drug management app and two congestive heart failure management apps using cognitive walkthroughs, heuristic analysis, and user testing. The study identified design problems that may affect senior persons’ usability, namely poor navigation, small button sizes, and insufficient data visualization. The study then proposed guidelines to help app developers design mHealth apps according to the cognitive, perceptual, physical, and motivational needs of senior persons.

In the context of Malaysia, it appears that most studies on mHealth have been general, with few specifically looking at senior persons’ needs. In terms of general mHealth studies, Lee et al. [36] collected data from 4504 Malaysian respondents on their mHealth and technology literacy. The results showed that only 20.4% of the respondents were aware of the term ‘mHealth’ or had used applications related to mHealth. The study also found that Malaysians have the intention to use mHealth apps in the future but mentioned needing more information and training on using these apps. This outcome suggests that mHealth in Malaysia is still in its early stage. Apart from that, Chew et al. [11] conducted a test on the usability and utility of the Med Assist app, which was developed to support ambulatory care patients in Malaysia. The results demonstrated that the app is user-friendly with multiple-user support and medication refill reminder features, which are considered very useful. In addition, Chong et al. [37] conducted a focus group discussion with 12 pharmacists regarding the development of an mHealth app in support of effective communication with deaf patients. The results provided feedback on the contents and design of the app, its potential benefits, and its usage challenges.

In terms of studies related to senior citizens, Salman et al. [13] identified four categories of usability problems related to smartphone user interface design for senior citizen. This was achieved by applying SMASH, a set of 12 usability heuristics, for smartphone and mobile applications within a controlled environment. Five experts with the necessary competence to perform the evaluation were enlisted. The experts’ prediction of usability problems was further tested among Malaysian senior citizens, revealing that 79% of senior citizens encounter the same issues identified by the experts. The findings confirmed that the four primary usability problems are appearance, language, dialogue, and information, each of which was given design solutions accordingly. Salman et al. [13] then developed guidelines for the creation of a refined prototype for senior persons. However, their study’s focus was on smartphone design in general and was not specific to mobile apps.

Based on above discussion, it appears that understanding senior citizens’ requirements is imperative for mHealth apps to be designed to meet their needs. Since there has been a dearth of research on the user requirements of senior citizens for mobile apps, there is scarce evidence to support their needs. In this regard, this study fills the gap by investigating the user requirements of Malaysian senior citizens for mHealth apps.

#### 1.1.6. User Acceptance and Usability Models

Several studies on mHealth have used adoption and acceptance models such as the Technology Acceptance Model (TAM), Theory of Reasoned Action (TRA), and Unified Theory of Acceptance and Use of Technology (UTAUT 1 and UTAUT 2) to understand the factors influencing the adoption of mHealth technologies. For instance, Wu et al. [38] employed the UTAUT 2 model to examine the factors influencing users’ electronic satisfaction (e-satisfaction) with mHealth apps and their continued usage behaviours towards the apps. Data was collected from 327 individuals who had used mHealth apps. The analysis found that the factors of performance expectancy, social influence, facilitating conditions, perceived reliability, price value, and online review positively influence users’ continued usage intention towards mHealth apps via the mediation of users’ e-satisfaction. Similarly, Samsuri et al. [39] developed an integrated framework using the TAM, TRA, and DeLone and Mclean model to understand users’ perceptions of a Malaysian mHealth app (MySejahtera). The analysis of data collected from 215 Malaysians revealed that system, service, and information quality have a positive influence on user satisfaction. It was further found that performance-related variables, such as content reliability, functionality, and performance expectancy, also increase user satisfaction, which in turn, promotes continuous usage of the app.

Usability-related models plays an important role in mHealth developments Co-design in technology development is a technique that systematically includes all key stakeholders so as to ensure that a product meets stakeholders’ needs and thereby, has enhanced usage [40]. According to Nielsen [41], usability is an important co-design factor; it refers to the ease of use of a system or resource, for which the device’s communication with the user is crucial. Devices are designed for certain uses and target audiences; usability refers to this specific purpose. User requirement studies have uncovered the information needed for co-design processes. Accordingly, several usability models have been developed to evaluate the usability of software or applications. For instance, Shackel [42] was one of the first to identify effectiveness, learnability, flexibility, and attitude as four important usability characteristics. Previous studies, such as those of Koohang and Du Plessis [43] and Thowfeek and Salam [44], have used Shackel’s usability framework to architect usability features into the e-learning environment. Shackel’s work was followed by Nielsen [45], who developed a model with five attributes that affect usability, i.e., efficiency, satisfaction, learning, memorability, and error. Subsequently, Wildenbos et al. [46] used this model to investigate age barriers in relation to mHealth application usage among senior citizens. Likewise, Muqtadiroh et al. [47] employed Nielsen’s usability model to study WikiBudaya, a cultural conservation system, wherein they employed the user testing method to assess usability quality.

Alternatively, Preece et al. [48] introduced throughput, learning, and attitude as the three major usability factors. Turner [49] adopted this model to investigate the simulation of five-a-side football using empirical modelling. On the other hand, the ISO 9241-11 [50] model identifies three different usability attributes, namely effectiveness, efficiency, and satisfaction. Green and Pearson [51] used this model to develop an instrument for web site usability, while Hussain et al. [52] employed it to evaluate a web-based health awareness portal within the mobile smartphone context.

Next, the ISO 9126 [53] provides an expanded model with five attributes, namely understandability, learnability, operability, attractiveness, and usability compliance. This model has been utilised in previous studies by Behkamal et al. [54] to evaluate business-to-business (B2B) applications that contribute to software quality as well as by Stefani and Xenos [55] to examine the role of e-commerce applications in improving the quality of customers’ interactive shopping experiences. Moreover, the PACMAD (People at the Centre of Mobile Application Development) model integrated the ISO and Nielson models to produce seven usability attributes: effectiveness, efficiency, satisfaction, learnability, memorability, errors, and cognitive load. Az-zahra et al. [56] adopted this model to investigate the usability of three of Indonesia’s most popular e-commerce applications (Tokopedia, Bukalapak, and Shopee), while Afif [57] used it to determine the extent to which university mobile applications’ usability qualities are attainable. Apart from these models, Stoyanov et al. [58] developed the mobile application rating scale (MARS) by combining indicators from previous research on user experience, technical aspects, and mHealth. The scale includes indicators such as aesthetics, engagement, app subjective quality, information quality, and engagement to measure the quality of an mHealth app. MARS has been translated into various languages, such as Arabic, Italian, German, Spanish, and Korean. For instance, Hee Ko et al. [59] tested the reliability and validity of its Korean version, MARS- K, proving that it is a reliable and flexible app quality rating scale that can be used by health professionals and app developers.

Based on the evolution of usability models discussed above, it is clear that assessing usability is crucial. It is a quality dimension that measures how usable an application is for different user profiles, such as the senior population in this study [60]. According to Mitzner et al. [61], some usability methods may not be suitable for mHealth apps with senior people as their target users. In fact, mobile developers find it difficult to manage the unique features of mHealth against the old age barrier in the usability measurement process [62]. Kurniawan et al. [62] thus emphasised the importance of developing mHealth apps with senior-friendly characteristics in order for the application to be used effectively by the ageing population.

Hence, given the significance of the usability model and its role in developing mHealth apps with senior friendly characteristics, we employed Nielsen’s [45] usability model as this study’s theoretical lens. Usability models with many dimensions are not always appropriate unless the dimensions are chosen based on user demands and interests or application functionality [63]. As a result, when addressing application functionality, carefully chosen dimensions are necessary to guarantee that a suggested model fits the needs of the mHealth application for senior users. In this regard, Nielsen’s five usability attributes (i.e., efficiency, satisfaction, learning, memorability, and error) are ideal and simple after removing redundancy and similarity with dimensions from other usability models [60]. Furthermore, Holzinger [64] considers Nielsen’s usability model to contain the most widely accepted attributes. This was confirmed by Liew et al. [65], who stated that Nielsen’s usability model is widely utilised in the evaluation of mHealth apps due to the applicability of its attributes.

Unlike Nielsen’s usability model, the ISO 9241-11 and ISO 9126 do not include learnability, memorability, or errors as usability attributes; nonetheless, they may be tacitly embedded in the concepts of effectiveness, efficiency, and satisfaction [62]. ISO models have the drawbacks of imprecise attribute design at the detail level, overlapping concepts, a lack of standard quality, insufficient assistance in analysing attribute findings, and uncertain attribute selection [66]. Hence, the ISO usability models were not selected in this study. Additionally, in comparison to Shackel’s and Preece et al.’s usability models, Nielsen’s is more advanced and comprehensive. Finally, the key contribution of the PACMAD model’s usability attributes is cognitive load, which refers to the amount of cognitive processing required by the user to use the system [67]. In contrast, senior users experience cognitive impairment that affects their daily functional abilities. This is caused by normal ageing and accompanying age-related diseases [68]. Furthermore, the cognitive load attribute of PACMAD was designed for users who will be performing a second action or dual task in addition to using the mobile application [67]. Senior people, conversely, choose to prioritise postural control over cognition in dual-tasking [69], which is consistent with traditional usability studies’ common assumption that users only perform a single task and choose to concentrate completely on that task [67]. As a result, the cognitive load attribute of PACMAD was regarded as irrelevant to senior users, who would likely be unaffected since they do not perform additional tasks while using the mobile device. Hence, upon comparing the usability models, we adopted Nielsen’s [45] usability model as the theoretical lens. Nielsen’s model provides a sense of structure that guided this study in identifying the requirements to develop a senior-friendly mHealth app. Table 1 illustrates the usability attributes adapted from Nielsen [45].

## 2. Materials and Methods

The qualitative approach was employed to understand the needs and expectations of seniors towards mHealth apps. Further, we identified user requirements and proposed a guideline for more engaging and senior-friendly mHealth apps. Therefore, the qualitative approach was suitable because the current study involved exploring the experience, meaning, and perception surrounding a phenomenon, which in this regard, was Malaysian seniors’ experience in and perceptions of using an mHealth mobile app. Second, the qualitative approach was deemed appropriate to obtain a deep understanding of the research topic [70].

To achieve the aim of this study, semi-structured interviews were conducted with a total of 74 Malaysian senior citizens (60 years old and above). The participants were aged between 60 to 84 and the average age was 68. The informants owned a smartphone and had experience using at least two to three mobile applications. They were recruited through several activities at religious premises within central Malaysia. Data collection took place at these religious premises after the activities. A few days before the activities, request letters were sent to the respective management of the religious places and approval was obtained to conduct interviews there. We relied on one of the four general criteria to trustworthiness by Lincoln and Guba [71]; that is confirmability. Confirmability is concerned with establishing that data and interpretations of the findings are clearly derived from the data and not based on particular preferences and viewpoints [72,73]. Hence, we constructed precision and accuracy in our research practice in this study to get as close to objective reality as the study can get [74].

Each interview session lasted from 45 to 60 min, during which audio recordings and field notes were taken simultaneously. A token of appreciation was given to the respondents following the interview sessions. The interview questions were designed using the Nielsen [43] usability attributes as a guideline. While the questions comprised the main attributes of learnability, errors, memorability, efficiency, and satisfaction, more information was gained during the sessions and the interview questions evolved as data collection progressed. Images of mobile app pages were shown to the interviewees to identify their preferences of text, colour, menu, background, and more. Questions related to participants’ mobile app usage and mode, type of health information retrieval, and preference of health management features were also included (see Appendix A Table A1). A larger number of participants (*n* = 74) were recruited in this study. This is because during the interview sessions, the participants were able to provide input related to app usages, preferred health management features, basic challenges while using apps etc., however some senior citizens were not able to provide detailed insights related to the various elements of usability attributes. Therefore, more participants were recruited to gather in-depth data on the usability attributes to design a comprehensive senior-friendly usability guideline.

To safeguard the informants’ interest and meet research regulations, approval was sought from the University of Malaya Medical Centre’s Medical Research Ethics Committee (MREC) prior to data collection. All the respondents were given an information sheet to inform them of the confidentiality and anonymity of their data. The respondents were also given a consent form to indicate their approval before data collection commenced. Interviews were voice-recorded with the permission and consent of the respondents. Some of the interviews were conducted in the Malaysian national language (Bahasa Malaysia), which were transcribed verbatim and then translated into the English language. The interview guidelines were translated by translators fluent in both the English and Malay languages. The translated guidelines were then reviewed by a reviewer who is also proficient in both languages.

In order to analyse the interview data, this study followed three concurrent flows of activity, as recommended by Miles et al. [75]. First, we conducted data condensation, where we selected, focused, simplified, and transformed the data in the interview scripts. Second, we organised the data and displayed the information using not only extended text but also tables and diagrams. The final stream of analysis activity was drawing conclusions. In terms of data analysis techniques, we employed the first cycle and second cycle coding processes [75], wherein we used descriptive coding for the first cycle and pattern coding for the second cycle. This process summarised segments of data prior to the identification of more general categories or classifications. Then, the categories or classifications derived from the analysis were mapped to the usability attributes proposed by Nielsen.

## 3. Results

### 3.1. Mobile Apps Usage

During the interviews, we attempted to find out the common mobile apps used by the informants. The main purpose was to comprehend how familiar they are with mobile apps. From the analysis of the interview data, it appears that most of the informants had experienced using several mobile apps, covering the areas of social networking, news, shopping, productivity, navigation, education, as well as health and fitness. Table 2 presents the common types of mobile apps used with relevant examples.

This suggests that a majority of the informants are able to recognise and use common functions in mobile apps, such as to login/logout, go to home, click on hyperlink, play video, listen to audio, enter information, and use common buttons (i.e., save, submit, cancel, and delete). They can navigate any mobile app well as long as they find the interface easy to use and engaging enough. However, the results demonstrate that the informants do not greatly desire interactivity and interactive functions in mobile apps. Most apps they currently use are merely for browsing, reading, and viewing purposes. One of the informants said, “It frustrates me if there are too many things (fancy UI) on the screen (mobile apps interface). I might not know where to find the thing (function/feature) I want to see. If it takes too long to find it, I will just skip using it” (Male, 66).

### 3.2. Challenges

Subsequently, we sought to further understand the informants’ concern about using mobile apps. From the analysis, we identified several challenges faced by seniors when using mobile apps. These challenges can be summarised into four categories, namely the apps’ usefulness, complexity, technical know-how, and necessity. Table 3 presents a brief description of these categories in the context of mobile app usage among senior citizens.

The first category is usefulness. Although a majority of the informants have significant experience using several mobile apps, they still feel that mobile apps are less useful and not highly applicable in their daily routine. To seniors, their day-to-day activities are not heavily affected with or without the mobile apps, especially given that the minimal number of errands they need to carry out a day. We then probed the informants with more questions on the available mobile apps for government services, which they can use from the comfort of their home. Apart from the fear of getting things wrong, the informants claimed they are used to interacting with humans (e.g., counter officers) at the premise. Therefore, they do not mind going to any local municipality when required.

The second challenge type is complexity. Most of the informants revealed their frustration towards interfaces that contain too much information, such as text, images and videos, hyperlinks, unlabelled icons, and tasks that require many steps. They tend to lose their focus and concentration when performing a task in mobile apps that have the abovementioned elements. We noted that some of the situations deemed complex by the informants can be regarded as simple by younger users. This situation may lead to seniors’ frustration and hesitance to use mobile apps, especially when there is limited assistance available. These circumstances are reflected in the following observations:

“At my age, I process information slowly. I wanted to learn how to use the technology, but it is difficult and complex to understand at times. I don’t know how to learn some of the things (functions/features) on my phone (mobile apps). So, I feel that it is difficult to use the apps.”

(Male, 68)

“My children taught me how to use the apps, but it doesn’t mean I can use the apps when they are not around. I can’t remember what they have taught me. They need to show me many times. But I can use apps for reading and browsing. Not the ones that require so many clicks.”

(Female, 73)

Third, seniors are challenged by technical know-how. The informants highlighted that they are unable to perform app installation on their mobile device to start using apps. They might require assistance to install, sign-up, and set up apps for the first time. Some of them do not have anyone, such as family members and relatives, to assist them. Hence, they might not want to use any mobile apps. Though we emphasised that most installations only require a few steps, the informants still feel that it is hard to execute because some steps require information processing and quick intake. Additionally, the informants find some error messages to be ambiguous, as they are not able to find the reason for the messages.

The fourth category of challenges is the necessity of the apps. There are many available mobile apps seniors can use for social interaction and better quality of life. Most of the informants expressed their liking for apps that support communication and leisure, such as Facebook, as well as apps that allow them to communicate with family and friends, such as WhatsApp. They feel that these apps are sufficient for them, since using too many apps could cause them to feel overwhelmed. To illustrate, “I enjoy using Facebook to keep in touch with old friends and family and we do communicate using WhatsApp. I hardly use apps for banking matters although I have the apps installed on my phone” said one informant during the interview.

### 3.3. Health-Related Information Seeking and Retrieval

**Figure 1:**
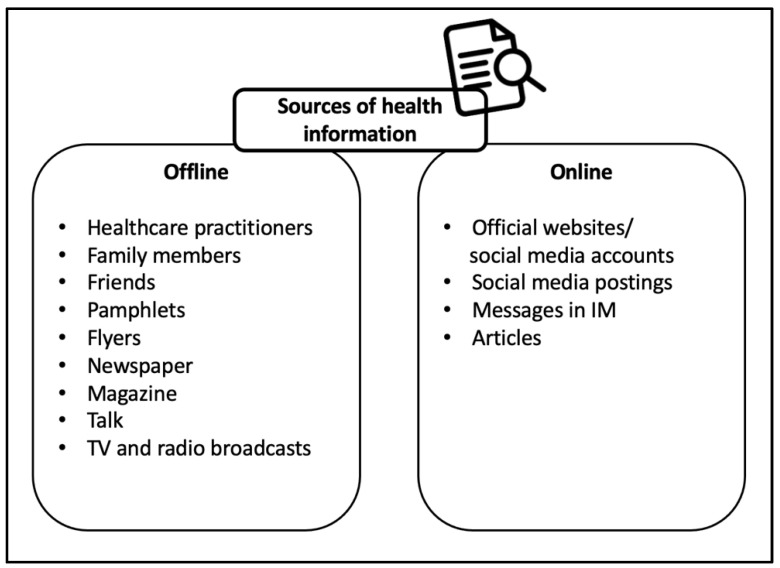
The sources of health-related information.

In earlier sections, we reported on senior citizens’ familiarity and concerns towards mobile apps in general. Subsequently, we investigated the informants’ perception and behaviour towards seeking and retrieving health-related information, especially on their mobile devices. A majority of the informants agreed that health-related information is extremely important. We identified several sources of health information, the most mentioned of which was healthcare practitioners (e.g., doctors, nurses, and physicians). Other sources of information are family and friends, pamphlets, flyers, articles, newspapers, magazines, social media posts, messages in instant messaging (IM), talks, as well as television and radio broadcasts. It can be said that most sources are from the offline mode (see Figure 1). These offline resources are easily accessible at hospitals, clinics, markets, public places, and religious sites and places.

The informants also claimed that printed materials are more readable as long they use larger fonts and appropriate typefaces. Only a small number of respondents had experience using web browsers to search for health-related information. This is mainly due to their limited knowledge on using search engines to retrieve appropriate information. It is also caused by trust issues, as senior citizens are concerned about the reliability of online information. It was observed that those who retrieve information online mainly access readily published information that appears on their social media timeline as posts shared by friends or posts from official pages they follow on Facebook. Some health-related topics that interest them on the Internet include diseases, symptoms and treatment, drugs and medication, diet plans, as well as alternative treatments.

### 3.4. Senior-Friendly Design for mHealth Apps

**Figure 2:**
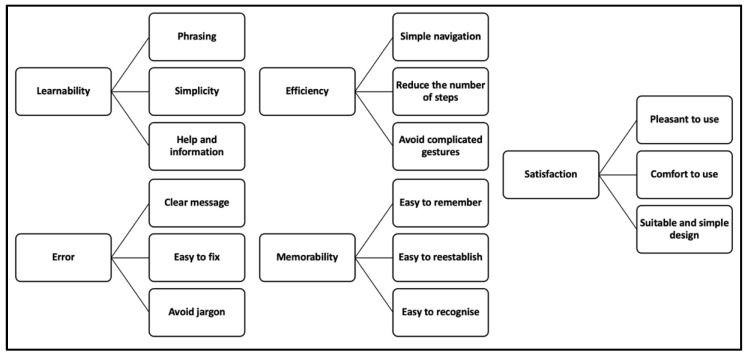
Nielsen’s usability attributes mapping.

The analysis of the interview data disclosed the important user requirements for a senior-friendly mobile application. The informants highlighted their preferences and expectations towards several items, such as menu, phrasing, font size, background, layout, colours, and more. We then mapped their preferences and expectations onto Nielsen’s usability attributes. The mapping is shown in Figure 2.

Based on these identified preferences and expectations, a guideline for a more engaging and senior-friendly usability design is summarised in Table 4 below.

### 3.5. Health Management Features

Although the informants raised several concerns about using mobile apps in general, they still feel that there is a need to use mHealth for a healthy lifestyle and the self-management of diseases. This is because they cannot fully rely on their caregiver or healthcare practitioners. Some of our informants stated that they do not have a dedicated caregiver and are unable to frequently visit hospitals for consultation or for retrieving required health information. Therefore, a majority of the informants agreed that using apps to access health information could help them manage their diseases and achieve a healthy lifestyle.

Apart from the proposed guidelines in the previous section, this section presents the possible important features that need to be incorporated into mHealth apps for seniors. The proposed guidelines, while considerably general, are closely applicable to mHealth. Additionally, this study proposes key health management features, as presented in Table 5. These features are deemed important in supporting seniors’ needs when using apps to maintain a healthy lifestyle and self-manage diseases.

## 4. Discussion

From the results noted, senior citizens are found to be more familiar with social networking apps, news apps, and shopping apps compared to apps for productivity, navigation, education, banking, and health and fitness. Seniors mainly used social networking apps to maintain contact with distant family members, friends, and acquaintances. This is consistent with the result of Wilson et al. [76] that senior people actively use social media to maintain relationships with their family and friends as well as to share or discuss common issues affecting them, such as financial or health issues.

Apart from social networking apps, senior citizens also commonly access news and shopping apps. They either have a news app installed on their mobile devices or read news from their mobile web browser that is accessed from their social media account. As for shopping apps, a number of them are already familiar with online shopping and enjoy its convenience. Nevertheless, they struggle to complete m-commerce transactions, and so require assistance from their family members in this aspect. These findings corroborate that of Msweli and Mawela [30], who highlighted that online transactions are challenging for senior persons, based on their systemic review of studies on the enablers and barriers of mobile commerce and banking among senior persons in developing countries.

To be able to develop mobile apps that fulfil the expectations of senior citizens, it is necessary to understand their concerns when using the apps. This study detected that the usefulness of mobile apps is one of the important factors for senior citizens when deciding to use an app. This outcome is also in line with Msweli and Mawela’s [30] finding that usefulness is a key enabler for the adoption of mobile technologies. It appears that senior citizens only use apps that prove to be useful and important to them. For instance, the senior citizens we interviewed are content with going to the bank to complete their financial transactions, even though mobile apps offer them the convenience of online banking. Further, seniors consider that learning and understanding how to use online banking is complex compared to making a trip to the bank itself. For them, some features are difficult to understand and too time consuming. For example, younger users may consider the “login” and “logout” process to be common in an app. Comparatively, senior citizens may not fully understand the purpose of those functions, which could worsen their intention to use the app.

Another challenge faced by senior citizens is their own lack of technical expertise in using mobile apps. They struggle to understand the technical issues and error messages that emerge when using the apps. This outcome was also noted by Wong et al. [8], whose study emphasised the negative impact of senior citizens’ low technical expertise on their understanding and resolution of technical issues when using mobile apps. For example, senior citizens have difficulties comprehending messages, such as insufficient storage. Moreover, the mobile device they use may not fulfil the minimum requirements for installing software or the server IP address may not be found. Complicated jargon may also frustrate senior users. Finally, the necessity of apps is another important issue because most respondents are of the opinion that they do not really need technology solutions in all areas of their life.

This study further noted that senior citizens mainly access health information from offline sources, such as printed materials. In contrast, using online channels proves to be a challenge due to difficulty reading from a screen. Their usage may also depend on the quality of the display device. Additionally, information overload and the reliability of health information available online can deter senior citizens from accessing health information online. This is because they tend to be more comfortable retrieving such information directly from their doctors. A small number of the informants mentioned that they do use online channels, mainly to read information shared by their friends on social media sites.

We also identified several important usability attributes of an elder-friendly mHealth app. The analysis showed that senior citizens prefer less technical terms and jargons in an app. Rather, they lean towards clear labels and titles and simple designs in apps’ registration forms. They also favour appropriate site maps that help them get to any page in the app, with suitable live hyperlinks that provide the needed information. All these features are related to the learnability attribute of Nielsen’s usability model [47].

Our analysis also indicated that senior citizens prefer simple language, appropriate typefaces, and larger fonts, as they have apparent difficulties reading text in small font. This result on font sizes aligns with Morey et al.’s [7] user testing of mHealth apps with senior citizens, which ultimately recommended using a larger font size of about 30 points for main titles or captions and at least 20 points for subtexts or subtitles. A similar finding also emerged in the work of Wildenbos et al. [9]. Therefore, it is suggested not only to have larger font sizes in mHealth apps, but also to provide options for users to adjust font sizes. That means mobile apps should offer users the flexibility to change their font face or font size based on user preference.

Similarly, simple step-by-step navigation should be encouraged in the app process, so that the number of steps involved in confirming messages is reduced, thereby saving time. In line with this, Morey et al. [7] stated that mHealth apps should not have more than three steps for entering data. By minimising steps, senior citizens’ confusion when using mHealth apps can be alleviated. When all these factors are incorporated into mHealth apps, usage by senior citizens would escalate because they would require less time to accomplish their tasks. Such an outcome would certainly minimise user effort, thereby aligning with the efficiency attribute of Nielson’s usability model [77].

Another observation was that mHealth meant for senior citizen should utilise appropriate and relevant photos and illustrations. These features are related to the memorability attribute of Nielson’s usability model, which can help seniors recall the options, app functionalities, and steps involved in app usage, particularly when revisiting apps after a certain span of time [47].

The informants of this study also commented that mHealth apps should provide clear instructions, especially in the process of making errors. These clear messages should guide them towards overcoming the issue faced. In addition, such apps should provide assistance in usage, for example, via tutorials on using the apps. Likewise, ‘frequently asked questions’ or FAQs can offer contact information to senior citizens to recover from their errors. This result is consistent with Wildenbos et al.’s [9] suggestion that feedback messages should be used in mHealth apps to guide senior citizens towards their next course of action and to help them recover from errors they make while using the app. These features contribute to the error attribute of Nielsen’s usability model, which underscored ‘easily recovering from the error encountered during app usage’ [12].

Finally, satisfaction is an important usability attribute. In this regard, aspects such as contrasting background, light colours, consistent layout and page design, and use of pull-down and cascading menus are preferred by senior citizens. These findings are consistent with Morey et al.’s [7] recommendation to have high text-to-background colour contrasts in mHealth apps to make it more convenient for senior citizens who have issues with declining vision. Besides the above features, mHealth apps need to incorporate the speech recognition feature too, so as to offer senior citizens more ease of use. Speech recognition helps turn speech into text, which acts as a virtual assistant to senior citizens by helping them complete the navigation process and retrieve the information needed. Through speech recognition features, users can easily use the ‘search’ function to perform their search within the app, secure information, request emergency assistance, and update information. These convenience-based and aesthetic components are related to the satisfaction attribute of Nielsen’s model, which gives pleasure to the user and increases their satisfaction with using the app [77].

Moreover, based on the feedback derived from senior citizens, we identified some important features that should be included in senior-friendly mHealth apps. The first among these is to include an SOS button for senior citizens during emergency situations. By just pressing a button, seniors should be able to call or send messages to a nominated emergency number. The second feature is to incorporate a medication reminder feature for taking the right medication at the right time, as senior citizens may suffer from memory loss or experience difficulty grasping complex medication regimes. Other health management features preferred by seniors include the management of medical records, appropriate diet plans, and relevant information on diseases, drugs, and clinical updates. Ultimately, these features help them self-manage their health activities. The above discussion has elaborated the details of senior citizens’ requirements and preferences to facilitate the development of an elder-friendly mHealth app.

In looking at the contributions of the current study, it has offered an understanding of senior users’ requirements from the mHealth app perspective. In this regard, this study contributes to the current literature on the usability of mHealth apps. Notably, we adopted Nielson’s usability attributes and found consistent results, most importantly, we proposed detailed “senior-specific” guidelines under each of these attributes. For example, one of the recommended features discussed earlier is enabling and improving a speech recognition feature. By turning speech into text, this feature is aimed to help senior citizens navigate apps and retrieve information using speech rather than typing. Moreover, based on the respondents’ inputs, we proposed relevant health management features to be incorporated in future mHealth apps to support the needs of senior citizens in managing their health and leading a healthy lifestyle.

The results derived from the current study contribute to resolving usability problems and challenges faced by senior citizens due to user interface design. The proposed user requirement guidelines and the recommendations of health management features help software developers create an elder-friendly app, which would encourage more senior citizens to tap into its benefits. These apps, when further enhanced, can provide senior citizens with a convenient way of managing their health activities, thereby granting them easy access to health information. This can subsequently improve their health literacy, which would lower hospitalisation costs [78] for seniors and their families.

The limitations faced by this study can be traced to its generalisability. Data for this study was mainly collected from senior citizens in the central Malaysian region only. Future studies should include a wider range of senior citizens from all states in Malaysia. This would offer a more comprehensive user requirement guideline for the development of mHealth apps for seniors. We also refrained from collecting data from caregivers, who play a vital role in caring for and monitoring the activities of senior citizens. Therefore, future studies should collect data from caregivers as they can provide valuable feedback and input in terms of physical activity monitoring and health management features. The input of healthcare professionals would also be useful. Thus, future research should consider interviewing this group of professionals so that their input can be used to further develop mHealth apps. More research on the design and implementation of speech technology for senior citizens should be conducted as well, as our results show that this feature is preferred by senior citizens due to its support in retrieving health information and facilitating self-care.

## 5. Conclusions

The growing prevalence of mobile application usage throughout the world has made the usability of mobile apps an emerging field of research. App development is challenging because each app has its own purpose to fulfil, whereas each user has specific needs and expectations from the app. This study has presented a compilation of senior citizens’ requirements for mobile apps, taking into account their existing concerns and difficulties. The study also detected the health information and health management features that are preferred by senior citizens. Based on the results, we proposed a user requirement specification that can guide software developers in the formulation of an elder-friendly mHealth application.

## Figures and Tables

**Table 1:** The usability attributes.

Usability Attributes	Description
Learnability	Users should be able to easily learn how to use the mobile app when they use it for the first time. Mobile app content should be easy to understand.
Errors (As in low error rate)	Less errors should be encountered by the user when using the mobile app and they should be able to recover from the error easily. Besides, to increase the understanding of the error messages, app should contain less complex error messages.
Efficiency	The mobile app should be efficient and assist user in completing the task in a timely manner. The app should contain features such as large icons, simple, clear, and short text display which improves efficiency
Memorability	The user should be able to recall the steps involved in using the mobile app after a certain period. For example, the app may include photos and illustrations to indicate or remind users in recalling the steps involved in the app usage.
Satisfaction	The app should be likeable, convenient and include aesthetic elements which increases the satisfaction of using the app.

**Table 2:** The common types of mobile apps used by the informants.

Types of Mobile Apps	Examples
Social networking	Facebook, Instagram, WhatsApp, Telegram, YouTube and Twitter
News	The Star Malaysia, Astro Awani, Berita Harian Mobile and Malaysiakini
Shopping	Lazada, Shopee, Mudah.my and Tesco Online Malaysia
Productivity	Gmail, Yahoo and Mail
Navigation	Google Map and Waze
Education	Bible, Quran
Health and Fitness	Fitbit

**Table 3:** The challenges and their descriptions.

Categories	Description
Usefulness	The degree for being useful or applicable in daily activities
Complexity	The degree for being intricate or complicated in performing tasks on screen
Technical know-how	The practical knowledge or skill to accomplish technical aspects in the apps
Necessity	The degree of being required in daily activities

**Table 4:** The user requirements guidelines.

Item	Guidelines
Phrasing	Uses active voice Uses positive phrasing in clear manner. Avoid too many technical terms and jargons
Menus	Uses pull down and cascading menu
Simplicity	Uses simple language for text or technical terms
Typeface and type size	Uses suitable typeface (e.g., San Serif) Ensure font is large enough and easy to read e.g., 12 or 14 point Allow users to change their preferred font face and size
Backgrounds	Uses a higher and good contrast between text and background (e.g., light text on dark backgrounds)
Layout	Uses consistent layout and standard page design
Colour	Avoid using bright colours (e.g., yellow and red)
Navigation	Uses same and simple navigation on each page. Uses step-by-step navigation procedures Offers users a choice of navigation options. Uses ‘breadcrumb’ style to support users with poorer short-term memory Reduce the number of steps and confirmation messages Avoid complicated gestures Fast response time
Icons and buttons	Uses versatile and simple symbols as icons Incorporate text with icons Uses large buttons and easy to tap with a finger
Forms	Uses simple design Provides clear labels and titles
Help and information	Provides a tutorial Offers contact information
Photos and illustrations	Use relevant photos
Site maps	Map the flow of the mobile app As reference for the users to get to any page in the app
Hyperlinks	Uses icons together with text Ensure live links are clearly separated from one another Reduce possibilities to accidentally clicking on the wrong adjacent link Avoid opening links in a new window as it may cause confusion
Error message	Provides a clear explanation State explicitly what needs to be done to solve the problem Avoid too many technical terms and jargons Make it easy to fix
Speech Recognition Feature	Turn speech into text Helps to navigate the app and retrieve information using speech rather than typing

**Table 5:** The health management features.

Feature	Description
Emergency	This feature allows users to avail medical assistance in an emergency with a touch of a button. Such assistance can be provided by ambulance services, caregivers or doctors who were assigned to the users.
Medical Records	This feature provides convenient tracking of users’ medical records. The medical records can be accessed from anywhere at any time with Internet access.
Reminder	This feature reminds and notifies users for important matters such as appointment with doctors, medication regimen and exercise routine in order to adhering to a healthy lifestyle.
Diet plan	This feature provides recommendations on diet plan for the users to ensure they reach their health and fitness goals.
Information on diseases	This feature is crucial as senior citizens need to keep abreast with the current clinical updates on the existing geriatric diseases and health problems.
Medical calculator	This feature is essential to be included in the app. The relevant health risks calculators for senior citizens includes mental health, respiratory health, vision and hearing, bone health as well as healthy lifestyle.

## Data Availability

The data presented in this study are available on request from the corresponding author. The data are not publicly available due to confidentiality and anonymity.

## Appendix A

**Table A1:** **Interview Guidelines.**

No.	Questions	Probes
**Section 1: Attitude towards technology and health app**		
1	What is your opinion about using the technology?	
2	Can you name the mobile app(s) that you are currently using?	
3	Which source do you consult for information concerning health?	Doctors, friends, family members, newspaper, magazines, online, etc.
4	Do you use any online sites (Google, YouTube, and Facebook etc.), mobile apps (any health app) as a source of information for health-related matters?	If yes, can you name them? How frequent do you use them?
5	Would you like to use a special health app for health management and health information access?	
6	What type of health information would you like to access from the health app?	information about any diseases, drugs or etc.
7	What type of health management features would you like to have in the health app?	Doctor Appointment Reminder Medication Reminder Diet Management Medical Records Storage Emergency button
**Section 2: Usability/Ease of Use of a general mobile app**		
8	What is your opinion about the mobile app that you have used or are currently using? i. Do you like the appearance of that app? ii. What feature(s) of the app attracts you to use it? iii. Are you ok with accessing the app by logging-in or do you want to directly go to the content? iv. Is it easy to type with keypads? v. Do you like the appearance of that app? vi. What feature(s) of the app attracts you to use it? vii. Are you ok with accessing the app by logging-in or do you want to directly go to the content? viii. Is it easy to type with keypads? ix. Was the content provided by the app useful? x. Are you able to launch the app and use it by yourself without anyone’s help? How long did it take to get familiar with the app? (Hint: Whether it was few days or months?)	
9	How do you normally navigate within the apps? i. Do you prefer slow response (time) for an action to take place or a quick response? ii. Do you prefer more explanatory text or simple messages? iii. Do you prefer vertical or horizontal scroll bars? Do you prefer alert/confirmation messages everytime you perform an action? Or only during some very sensitive actions like closing the app or terminating a process?	
10	Do you think that only you should have permission to access or update your details? Or your partner or children can have access to monitor or update your details?	
11	Could you please describe the common difficulties you encountered when using mobile apps?	What are the complex functionalities? Lack of clarity of the content. Lack of clarity of the language used. After using the app for once or for few times, have you discontinued using it? If yes, what is the reason?
12	Could you please describe the preferences in term of visual aids (e.g.,: backlight, size of text, style, bold color, color scheme and size of buttons/icons, etc.) i. Do you prefer the presentation of text in colour or black and white? ii. Do you like bright or mild color background for the apps? iii. Do you prefer the buttons to be identified with text or images? Which will be easier to remember? iv. What is the common text size preferred by you? Do you like to have the zoom in, zoom out facility to magnify and minimize the content?	Will that be useful? Or will it be a problem as it may sometimes resize the content accidentally when touched.
13	Have you ever used the speech recognition and text-to-speech features in your mobile phone or in any mobile apps?	If yes, explain about your general experience in using the speech technology. Did you face problems with voice input?
14	Would you like to use the speech recognition and text-to-speech features in the health app? i. Do you like to retrieve health information through speech? ii. Setting or updating the health-related data through speech. iii. Getting emergency help through speech. For any other tasks would you like to use speech rather than typing?	If yes, how do you want the speech technology to help you in the health management aspect?
15	Do you like to give any other suggestions to the developers for the new health management app for seniors?	

Note: This is a brief version of the interview guidelines. The actual guidelines used for the interview consist of a number of images to assist the interview session.
